# Supplementary material for: Differential systemic inflammatory responses after TAVI: The role of self versus balloon expandable devices
Source: PLoS One. 2021 Oct 26;16(10):e0258963. doi: 10.1371/journal.pone.0258963 (PMC8547641; doi:10.1371/journal.pone.0258963)
Supplement: Attachment — Submitted filename: review opinion.docx [file pone.0258963.s001.docx]

In their manuscript titled “Differential systemic inflammatory response after TAVI: The role of self versus balloon expandable devices”, authors tried to compare SIRS between SEV and BEV. The conclusion revealed (1) SEV trigger more SIRS than BEV groups and (2) the short term effect may be associated with long term valvular durability. However, the whole tables did not show any comparison between SEV and BEV, except table 1 (demography). The remaining table just manifested total patients result. Reader may more interest in comparison of WBC, absolute neutrophils, absolute, lymphocytes, postop complication, and short-term result between SEV and BEV groups. Although authors mentioned the difference between two groups in Figure 3, there is no comparative result to enhance the effect of SIRS between two groups, such as postop complication, short-term result. These comparative results should be listed in tables so that matched their title “SEV vs BEV”. Second, short-term result was shown on table 4 and longer result was on Figure 4 (KM curve). The big problem is table 4 is calculated from total number but figure 4 statistics is from separated group. So, I am not sure the authors can conclude “the short-term effect may be associated with long term result”. Furthermore, there isn’t any postop valve evaluation result in this article. This conclusion didn’t make sense.

As authors’ mention, any implant will induce SIRS in patients. So, if authors just show total patients’ data in WBC, neutrophil, lymphocyte, N/L ratio change after TAVI without separated groups’ comparison, it is very usual and predictable result. Many aortic stent graft paper also discuss about effect of metal material and SIRS because stent graft material is very similar to TAVI’s valve support structure, especially in metal material. So, this article should classify the total patients and focus on cobalt chromium (BEV) and nitinol (SEV) comparison. Try to discuss “different” metal material to cause “different” prognosis by “different” SIRS results and statistics.

Generally speaking, this title didn’t match their result and this result (total patients) is also not special. Study of different metal material induce SIRS is frequently published in the patients who receiving aortic stent graft procedure. Please classify to two groups by BEV and SEV groups more detail. Analyze individual pre-, post- inflammation index, perioperative parameter, postoperative complication, short-term follow-up, and valvular durability so that authors can conclude “The role of self versus balloon expandable devices”

Some questions are still required to clarify:

Major issue

1. In Materials and Methods, “We excluded patients……….those with periprocedural death (up to 72 hours after TAVI)”, why you choose 72 hours as your exclusion criteria but not 24 hours? 48 hours? 96 hours or even one month?? Could you offer any reference???
2. This paper discussed about inflammation response after TAVI. Although their TAVI protocol mentioned about Aspirin 100mg before procedure and dual-antiplatelet with Aspirin after that, some patients may use Aspirin, anti-platelet agent, or statin for their long-term underlying disease before procedure. Those medication can inhibit different inflammatory pathway level. So, please list patient medication in the demography and compare whether it is significant difference between two groups. Medication may affect postoperative SIRS result.
3. How do authors select BEV or SEV methods for the patient treatment?? Is there any criteria?
4. In table 3, it is very disorganized. Author may try to identify which factors (variables) may influence inflammatory markers after TAVI. However, they should organize these variables by different statistics methods. Some variables are continuous but some are categoric. If X and Y (inflammation markers) are continuous variables, please use linear regression and organize as one table. Some X belongs to categoric (hypertension, DM, TAVI type…….), authors may consider to classify inflammation markers (Y variables) to binary variable by certain cut point and use univariate logistic regression to make another table. In this manuscript, if authors can prove TAVI type (SEV and BEV, categoric variables) is one factor for influence of inflammation markers (have been classified to binary variables), that will be very significant finding and match this manuscript’s title. Anyway, separate to two tables by different statistics methods may be easier for reader understanding.
5. In table 3 again, if contrast volume and time may be the factors to influence inflammatory markers, TAVI type should be evaluated with the two factors together by multivariate logistic regression to make sure whether TAVI type (SEV and BEV) is associated with inflammatory marker changes.
6. In discussion, it may have to be re-written because manuscript result can’t support authors’ conclusion enough. Most of discussion sentences were written or quoted by other paper result. I will suggest author reorganize whole article after recalculating convincible tables and figures so that authors can say something to correspond their title.

Minor issue
No
