# Supplementary material for: Differential systemic inflammatory responses after TAVI: The role of self versus balloon expandable devices
Source: PLoS One. 2021 Oct 26;16(10):e0258963. doi: 10.1371/journal.pone.0258963 (PMC8547641; doi:10.1371/journal.pone.0258963)
Supplement: Attachment — Submitted filename: Response to Reviewers FR.docx [file pone.0258963.s002.docx]

Cardiology Division

Affiliated with The Hebrew University of Jerusalem

31 May 2021

## Emily Chenette, Editor-in-Chief

### Sara Fuentes Pérez, Senior Editor

### PLoS ONE, Cardiovascular and Public Health

Detailed answers to the editor and referees comments:

1) *Survival rates are similar between patients with SEV and BEV, suggesting the minimum impact of TAVI-induced inflammatory response on the survival. Relevance of increased WBC and neutrophils only 24 hours post procedure should be provided. At a minimum, discuss the relevance more in detail.*

**Despite the statistically significant differences between the two types of valves at 24 hours post-procedure, this is not reflected in the survival rate. This is similar to other clinical situations where laboratory significant changes are seen without a clinical translation. Similarly, thrombocytopenia after TAVI is a universal and virtually inevitable phenomenon in more than 90% of the patients. However, a clinical impact is only found when there is an extreme thrombocyte count drop.** **In our present study, we did not discriminate according to the inflammatory response intensity and the clinical outcomes , which probably can shed light this finding.**

(These sentences were added to the main manuscript, Discussion section)

2) *Describe the selection process for BEV and SEV.*

**The type and size of the valves were at the discretion of the local heart team after they assembled, and a decision was made according to the patient’s anatomical and clinical characteristics.**

**(This sentence was added to the main manuscript, Methods section)**

3) *Provide information on medications including statins, other lipid lowering drugs, and anti-diabetic drugs.*

**See new Table 1. We added the drug history for each patient which includes antiplatelet, anticoagulant, lipid-lowering agents, steroids, and medications for diabetes and hypertension.**

4) *Consider potential confounders, including medications.*

**Some confounders, such as the peri-procedure use of anti-inflammatory drugs, including steroids in iodine contrast allergy mitigation was minimal and with a similar rate in the two groups, thus there is no bias.**

(This sentence was added to the main manuscript, Discussion section)

5) *Provide more detailed methods, including determination of study size and definition of diagnosis (hypertension, diabetes, dyslipidemia, smoker, atrial fibrillation, coronary artery disease, etc) and all outcomes (bleeding, major vascular complication, stroke, myocardial infarction, AKI, and arrhythmia).*

We performed a retrospective study, including all the patients **that** underwent TAVI in **a** 10-year period**.** Therefore, this is not an event rate driven study.

**The definition of the patients’ baseline characteristic risk factors included the corresponding diagnosis in the medical records together with the chronic use of anti-hypertensive, glycemic control and lipid lowering drugs, respectively.** **Coronary artery disease (CAD), atrial fibrillation, peripheral arterial disease and strokes, were based on the medical records and confirmed by complementary clinical tools, such as angiograms in CAD. All the outcome definitions were strictly determined according the Valve Academic Research Consortium 2 Criteria (VARC-2).**

(This sentence was added to the main manuscript, Methods section)

6) *Describe the time point when inflammatory markers (blood samples) were obtained post TAVI; 24 hr 72 hr or 6 months, in Table 3*

See the corrected new Table 3**.** We considered inflammatory markers at 24 hours.

7) *Explain the data on table 3 and 4 more in detail with the method of statistical analysis in the main text and table legend*

We add**ed** the two groups of (yes) and (no) for each variable and we d**id** the comparison.

**Data are presented as means ± standard deviations. Continuous variables between the various study groups were tested for normality by a Shapiro- Wilk test and when an abnormal distribution was found, a Mann- Whitney test was performed. When the distribution was normal, a t-test was used.**

(This sentence was added to the main manuscript, Methods section and the table legends were changed)

8) *Describe the method of statistical analysis in Figure 3.*

**Repeated measures analysis of variance were used to determine any significant differences between variability over time between SEV and BEV.**

(This sentence was added to the main manuscript, Methods section)

9) *Unites are missing in a lot of data. Provide.*

We **inserted** **all** missing units.

10) *Number at risk should be provided in Figure 4*

This parameter was add**ed** to the new Figure 4.

11) *Provide more detailed figure legends. Also, add figure 4 legend*.

All Tables and Figures legends were changed

Detailed answers to all the referee comments:

Reviewer #1:

1. *In Materials and Methods, “We excluded patients……….those with periprocedural death (up to 72 hours after TAVI)”, why you choose 72 hours as your exclusion criteria but not 24 hours? 48 hours? 96 hours or even one month?? Could you offer any reference???*

We excluded these kind of patients due to the lack of blood samples and follow-up.

2. *This paper discussed about inflammation response after TAVI. Although their TAVI protocol mentioned about Aspirin 100mg before procedure and dual-antiplatelet with Aspirin after that, some patients may use Aspirin, anti-platelet agent, or statin for their long-term underlying disease before procedure. Those medication can inhibit different inflammatory pathway level. So, please list patient medication in the demography and compare whether it is significant difference between two groups. Medication may affect postoperative SIRS result.*

We added in Table 1 the patients**’** chronic medication list and the use of steroids in iodine allergic patients just before the procedure. Also**,** we added in the Discussion section**,** a reference to the drugs (see point 4 above)**.**

3. *How do authors select BEV or SEV methods for the patient treatment?? Is there any criteria?*

See point 2 above**.**

4. *In table 3, it is very disorganized. Author may try to identify which factors (variables) may influence inflammatory markers after TAVI. However, they should organize these variables by different statistics methods. Some variables are continuous but some are categoric. If X and Y (inflammation markers) are continuous variables, please use linear regression and organize as one table. Some X belongs to categoric (hypertension, DM, TAVI type…….), authors may consider to classify inflammation markers (Y variables) to binary variable by certain cut point and use univariate logistic regression to make another table. In this manuscript, if authors can prove TAVI type (SEV and BEV, categoric variables) is one factor for influence of inflammation markers (have been classified to binary variables), that will be very significant finding and match this manuscript’s title. Anyway, separate to two tables by different statistics methods may be easier for reader understanding.*

According to the reviewer’s comments, we clarified and consequently changed this table format.

5. *In table 3 again, if contrast volume and time may be the factors to influence inflammatory markers, TAVI type should be evaluated with the two factors together by multivariate logistic regression to make sure whether TAVI type (SEV and BEV) is associated with inflammatory marker changes.*

The study focused on the inflammatory response. We have done a multivariable analysis for these items, but we considered them to be not relevant to this article.

6. *In discussion, it may have to be re-written because manuscript result can’t support authors’ conclusion enough. Most of discussion sentences were written or quoted by other paper result. I will suggest author reorganize whole article after recalculating convincible tables and figures so that authors can say something to correspond their title.*

As a result of all the major changes requested by the editors and four rewrites, the article was significantly changed, mainly in the discussion section. We wrote an original paper that included ideas and conclusions from the literature to support or refute our hypothesis which is findings based*.*

Reviewer #2:

1. *For instance no data on use of antibiotic treatments are provided*

The use of prophylactic antibiotics before or after TAVI procedure is not routinely recommended (Gomes B, *Periprocedural antibiotic treatment in transvascular aortic valve replacement*. J Interv Cardiol. 2018 Dec;31(6):885-890). To clarify this point, we added a sentence to the Methods section of this manuscript:

**The use of prophylactic antibiotics during the procedure or hospital stay was routinely avoided.**

2. ” other markers of inflammation such as C - reactive protein or procalcitonin should have been taken into consideration”.

The CRP values measured in 62 patients was 3.2 ± 4.2 mg/L at baseline and 9.8 ± 8.1 mg/L 24 hours after the procedure (p < 0.001). These values were obtained in a minority of the patients and were not performed in all the timeline sequences (at admission, after 24, 48, 72 hours, and at 6 months). We did not do any procalcitonin assays. Therefore, we did not include this data in the manuscript. If the reviewer or editors insist, we can add this point in the study limitations paragraph.

3. *In the discussion the passage on leaflet components spares any support by the data and remains hypothetical*

According our findings, the inflammatory response quantified by neutrophils, lymphocytes and N/L ratio, is greater for SEV compared to BEV. The differences in the materials for the components, design and deployment system can hypothetically explain this significant finding.

Reviewer #3:

1. *Do the authors have monocyte data as well?*

All the monocyte behavior are given as neutrophils and lymphocytes. As you can see in Table 2 and Figure 2, included in the new article version, the findings are amazing as the other components described in the manuscript. We have not given any explanations for this finding because monocytes are not part of the SIRS response and we did not perform an assessment of their activation status, a crucial point when analyzing their possible pathophysiological role.

**As recently described by Baratchi et al., Circulation. 2020;142:1092–1105, monocytes have a pivotal role in the chronic process and contribute to aortic stenosis development. We found a significant rise in the monocytes count in the acute phase, but we did not measure their activated function. This limits the possible interpretations.**

(This sentence was added to the main manuscript, Discussion section)

Reviewer #4:

1. *I do not agree with or do not understand the authors’ discussion statement: “The addition of NLR to the TAVI risk score improved the predictability of MACE after TAVI.” What ‘TAVI risk score’ are the authors referring to?*

We agree that including our findings in the weighting of existing TAVI scores such as the French CAPRI, American TAVR or UK scores, was not explained enough.

**The procedure risk score assessment is a dynamic process, it will change according the clinical evolution. We propose to add our findings, as they are inexpensive to do, to the well-established and known pre-TAVI procedure scores, such as CAPRI.**

(This sentence was added to the main manuscript, Discussion section)

2. *Why was pre-TAVI septal hypertrophy associated with a higher risk of postprocedural neutrophilia? Possible explanation?*

This finding is interesting and relatively surprising. One hypothetically explanation can be based on the hemodynamics and shear stress in the setting of a severe aortic stenosis. Because the scope of the present study focuses on inflammation response, we did not develop this finding.

3. *Please explain/shortly discuss the association between the VARC-2-defined 30-day outcomes and WBC elevation? Why was leukocytosis associated with increasing bleeding rates?*

**The association between unfavorable 30 day outcomes of deaths, bleeding and vascular access complications, according to the VARC-2 definition, highlights that the more complex procedures provoke major inflammatory responses. Therefore, less favorable outcomes or inherently, a major inflammatory response predisposes the patient to unfavorable clinical events related to the existing milieu.**

(This sentence was added to the main manuscript, Discussion section)

.

|  | **ד"ר אלכס בלאט**  רופא מנהל שירות אשפוז  [alexbl31@clalit.org.il](mailto:alexbl31@clalit.org.il)  מצנתר בכיר \| מערך קרדיולוגיה  972-8-9441335 \| 972-8-9444628 \| fax | **Alex Blatt MD, MSc**  Senior Cardiology  [Ab[alexbl31@clalit.org.il](mailto:alexbl31@clalit.org.il)](mailto:Abcdefg@clalit.org.il)  Interventional Cardiology \| Cardiology Department |
| --- | --- | --- |
