# Supplementary material for: Differential systemic inflammatory responses after TAVI: The role of self versus balloon expandable devices
Source: PLoS One. 2021 Oct 26;16(10):e0258963. doi: 10.1371/journal.pone.0258963 (PMC8547641; doi:10.1371/journal.pone.0258963)
Supplement: Attachment — Submitted filename: review opinion 2.docx [file pone.0258963.s003.docx]

In their manuscript titled “Differential systemic inflammatory response after TAVI: The role of self versus balloon expandable devices”, authors tried to compare SIRS between SEV and BEV. In the revised version, author still didn’t respond several reviewer’s question accurately. Although the most critical group outcome have been shown (table 5), it didn’t reveal any significant different between SEV and BEV. Hence, it means 24 hr inflammation response difference (Table 3 and Figure 3) can’t affect the outcome between SEV and BEV according to Figure 4 and Table 5. This paper just can say authors found higher inflammatory response in SEV but can’t arbitrarily conclude this difference is associated postoperative outcome, either short-term or long-term. In other words, operator may not worry about postoperative care even higher inflammation response in SEV was found clinically because the outcome is similar to BEV.

Meanwhile, author used a lot of previous paper to explain higher inflammation response may get poor result in discussion. However, these previous evidences can’t correspond and echo this paper because SEV and BEV didn’t show significant difference in postoperative outcome in current data (Table 5). The better discussion for the result of this paper is about why higher inflammation response was found in SEV instead of outcome comparison. In current revised discussion, author mentioned about the material difference between SEV and BEV, including metal part and bovine valve decellularization. They should list more factors may affect inflammation response, such as fibrinogen level, D-dimer level, nitinol concentration and so on. That will be more reasonable to the topic of this paper.

The question as below:

1. In table 2 and table 3, authors listed 24hr WBC, neutrophil, lymphocyte, and NLR. However, the values are a little different (ex: WBC 10.08±3.55 in table 2 vs 10.101±3.671). Do they calculate from the same groups???
2. In discussion: “Our results are in agreement……..which probably can shed light this finding” may be deleted.

Although authors want to focus on the inflammation response, they described too many outcome-related sentence in the discussion. Please remove these sentence and match original result to avoid readers confusion.

3. In discussion, “To the best of our knowledge…….heightened inflammatory response as compared to the BEV system” is good and reasonable to paper result. If author can list the table compare with nitinol concentration, D-dimer, fibrinogen…..so on between SEV and BEV, these biochemistry data will be more convincible and can more focus on inflammation response. That will be the real first report.
4. How do authors select BEV or SEV methods for the patient treatment??? Authors didn’t answer directly. The selection bias may associate post-inflammation response. Please describe and clarity.
5. Contrast and surgical time are very significant difference after 24hr postoperatively to preoperatively. Contrast amount and prolonged surgical time may induce cytokine secretion. Please compare with contrast and surgical time between SEV and BEV. It may explain the inflammation response different between groups.
6. What about prior aortic valve replacement surgery between SEV and BEV (valve-in-surgical valve)??
7. How many valves were used (rescued) during surgery if the first deployed valve was malfunctional (valve-in-valve)??
8. What about reintervention rate between two groups (valve-in-valve)??
