# Supplementary material for: Differential systemic inflammatory responses after TAVI: The role of self versus balloon expandable devices
Source: PLoS One. 2021 Oct 26;16(10):e0258963. doi: 10.1371/journal.pone.0258963 (PMC8547641; doi:10.1371/journal.pone.0258963)
Supplement: Attachment — Submitted filename: Cover letter_Response V3 FR.docx [file pone.0258963.s004.docx]

Cardiology Division

Affiliated with The Hebrew University of Jerusalem

6 October 2021

## Emily Chenette, Editor-in-Chief

### Sara Fuentes Pérez, Senior Editor

### PLoS ONE, Cardiovascular and Public Health

Dear Profs. Chenette and Fuentes Pérez,

Thank you for the opportunity to revise our manuscript. We appreciate the acceptance of all previous corrections that have been submitted as you requested and elevating the quality of study. We have made significant efforts to meet the additional reviewer and editor comments.

Attached is a point-by-point letter addressing the comments and the changes made accordingly.

We are submitting (uploading) the revised new manuscript version and deleting the previous file. The new sentences have been placed as described in this letter below. The sentences in **bold** type are the updated changes.

We hope you will find the current version and the changes made, worthy of publication in the *PLoS ONE* Open Journal.

Sincerely yours,

Alex Blatt MD, MSc

Detailed answers to the editor comments:

1) In the abstract, limitation regarding a lack of long-term effect on inflammation and outcomes should be added, such as “long-term effect needs to be investigated” or “clinical studies are warranted to assess the long term effect”.

This sentence was added to the abstract, Conclusion section.

2) The conclusion in the abstract, “this short term effect may be associated with long term valvular durability” is not supported by your data and should be deleted.

This sentence was deleted.

3) The sentence, “an increase in WBC … was associated with higher rates of major …” (result section (clinical outcomes) and the sentence, “this response was also found to be associated with a worse 30 days outcome” (Discussion (first paragraph) should be de-emphasized since there is no difference or tendency in 30-day outcome (table 5).

The order of the sentence in the Discussion section, first paragraph, was inadequate and lead to confusion. Findings from table 4 and 5 wrote intercalates, provoke confusion. Now, we order in a more clear version.

4) Small sample number should be included in the limitation.

We added a paragraph to describe the study limitations, including sample size.

Detailed answers to the reviewer #1 comments:

1. In table 2 and table 3, authors listed 24hr WBC, neutrophil, lymphocyte, and NLR. However, the values are a little different (ex: WBC 10.08±3.55 in table 2 vs 10.101±3.671). Do they calculate from the same groups???

The minuscular differences in the blood cell count related to the drop down of two patients excluded in table 3 because lack of 6 month follow-up lab values.

2. In discussion: “Our results are in agreement……..which probably can shed light this finding” may be deleted.

This extended paragraph analyzed the core of our study integrated with the current published bibliography in the field. We see a legitimate discussion to include in the article.

3. In discussion, “To the best of our knowledge…….heightened inflammatory response as compared to the BEV system” is good and reasonable to paper result. If author can list the table compare with nitinol concentration, D-dimer, fibrinogen…..so on between SEV and BEV, these biochemistry data will be more convincible and can more focus on inflammation response. That will be the real first report.

We have limited values of these propose parameters. Nitinol concentration was values were not measured at all.

4. How do authors select BEV or SEV methods for the patient treatment??? Authors didn’t answer directly. The selection bias may associate post-inflammation response. Please describe and clarity.

This requires was answered previously.

5. Contrast and surgical time are very significant difference after 24hr postoperatively to preoperatively. Contrast amount and prolonged surgical time may induce cytokine secretion. Please compare with contrast and surgical time between SEV and BEV. It may explain the inflammation response different between groups.

In table 1 we demonstrated non-significant differences in the injected contrast material and procedural time between SEV and BEV.

6. What about prior aortic valve replacement surgery between SEV and BEV (valve-in-surgical valve)??

We performed V-in-V in a few number of patients, 7 in total.

7. How many valves were used (rescued) during surgery if the first deployed valve was malfunctional (valve-in-valve)??

Rescue additional valve implantation migration related was performed in a small number of patients, 6 in total.

8. What about re-intervention rate between two groups (valve-in-valve)??

Again, the patient needed re-intervention after TAVI is very small.

|  | **ד"ר אלכס בלאט**  רופא מנהל שירות אשפוז  [alexbl31@clalit.org.il](mailto:alexbl31@clalit.org.il)  מצנתר בכיר \| מערך קרדיולוגיה  972-8-9441335 \| 972-8-9444628 \| fax | **Alex Blatt MD, MSc**  Senior Cardiology  [Ab[alexbl31@clalit.org.il](mailto:alexbl31@clalit.org.il)](mailto:Abcdefg@clalit.org.il)  Interventional Cardiology \| Cardiology Department |
| --- | --- | --- |
